# *Drosophila *KDM2 is a H3K4me3 demethylase regulating nucleolar organization

**DOI:** 10.1186/1756-0500-2-217

**Published:** 2009-10-23

**Authors:** Harsh H Kavi, James A Birchler

**Affiliations:** 1 Division of Biological Sciences, University of Missouri, Columbia, MO-65211, USA

## Abstract

**Background:**

CG11033 (dKDM2) is the *Drosophila *homolog of the gene KDM2B. dKDM2 has been known to possess histone lysine demethylase activity towards H3K36me2 in cell lines and it regulates H2A ubiquitination. The human homolog of the gene has dual activity towards H3K36me2 as well as H3K4me3, and plays an important role in cellular senescence.

**Findings:**

We have used transgenic flies bearing an RNAi construct for the dKDM2 gene. The knockdown of dKDM2 gene was performed by crossing UAS-RNAi-dKDM2 flies with actin-Gal4 flies. Western blots of acid extracted histones and immunofluoresence analysis of polytene chromosome showed the activity of the enzyme dKDM2 to be specific for H3K4me3 in adult flies. Immunofluoresence analysis of polytene chromosome also revealed the presence of multiple nucleoli in RNAi knockdown mutants of dKDM2 and decreased H3-acetylation marks associated with active transcription.

**Conclusion:**

Our findings indicate that dKDM2 is a histone lysine demethylase with specificity for H3K4me3 and regulates nucleolar organization.

## Background

The recent discovery of Histone Lysine Demethylases containing the Jumonji domain has added an additional dimension to gene regulatory circuits [1]. The removal of histone lysine methyl modifications by JmjC domain demethylases finely calibrates gene expression. The JmjC group of demethylases interact with various silencing and activator protein complexes to regulate gene expression [2,3]. The biological role of histone lysine demethylases is largely unexplored in *Drosophila*. This study investigates dKDM2, which is an F-box protein (FBXL 10) in *Drosophila*.

## Results and Discussions

KDM2B was one of the first identified JmjC domain containing demethylases and was shown to exhibit demethylases activity towards H3K36me2[1]. KDM2B was also shown to act as a transcriptional corepressor and as a part of the PcG silencing complex[2,3]. A recent report implicated KDM2B as a H3K36me2 demethylase in regulation of cellular proliferation and senescence [4]. Another study conducted in human cell lines demonstrated that KDM2B regulates transcription of ribosomal genes and the structure of the nucleolus[5]. The same study found by protein over expression and immunofluoresence techniques that the specificity of the enzyme targets H3K4me3 and not H3K36me2 as reported previously. The fly homolog of KDM2B is the gene CG11033 (Flybase). The fly protein contains the Jumonji domain, an F-box degradation domain, a Zn finger domain (found in many chromatin associated proteins) and a leucine rich repeat region (plays an important role in protein-protein interaction). BLAST analysis of CG11033 genomic sequence revealed a consensus nucleolar localization motif rich in arginine and lysine (Additional File 1). KDM2B in humans also possesses the characteristic NoLS (nucleolar localization signals) and is present in the nucleolus[5].

**Figure 1:**
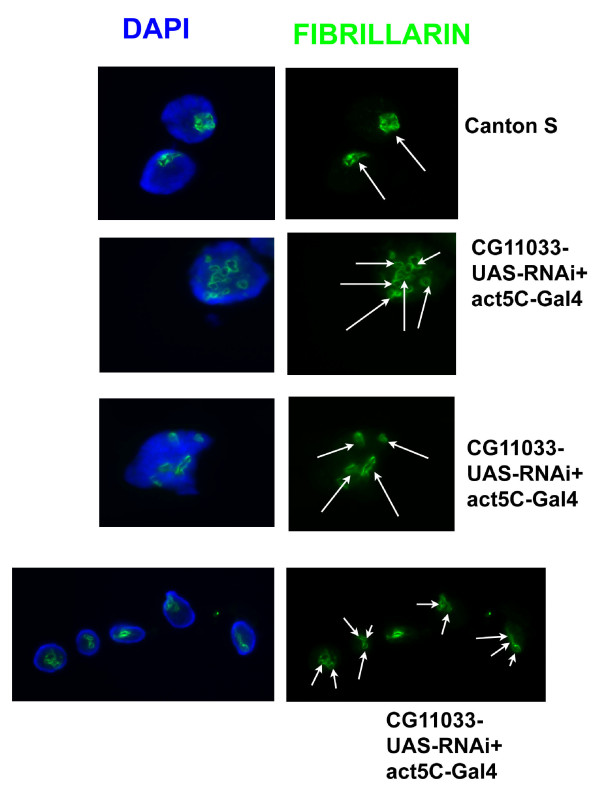
**Multiple nucleoli in the RNAi knockdown of KDM2 (CG11033)**. Arrows indicate fibrillarin spots.(nucleolus marker). Canton S was used as a control. Fibrillarin is used for staining the nucleolus in the gently squashed polytene nuclei.

The presence of the NoLS sequence prompted us to investigate the effect of CG11033 mutation on *Drosophila *nucleolar organization. Transgenic flies expressing inverted repeats of the CG11033 coding region under the influence of the UAS promoter were crossed with act5C-Gal4 flies. The presence of Gal4 leads to transcription of inverted repeats under the influence of UAS promoter bearing Gal4 binding sites. Flies bearing only the inverted repeats were used as a control. The expression of dsRNA arising out of transcription of inverted repeats brings about downregulation of CG11033. These mutants exhibited multiple nucleoli, which are smaller in size (Figure 1). A majority of them showed about 2-3 nucleoli while a few of them showed multiple nucleoli 4-7 (Figure 1 and Additional file 2). The controls (where dsRNA is not formed due to the absence of Gal4) showed a single punctuate nucleolus (Figure 1). The nucleoli were visualized by using fibrillarin antibody. Fibrillarin is a nucleolar marker and is important for rRNA maturation. Quantitative real time PCR analysis showed reduction of dKDM2 transcript level relative to tubulin mRNA level in RNAi knockdown larvae (Additional file 3). There was no change in the transcript level of *lid *(another H3K4me3 demethylase) mRNA in 3^rd ^instar larvae (Additional file 4).

**Figure 2:**
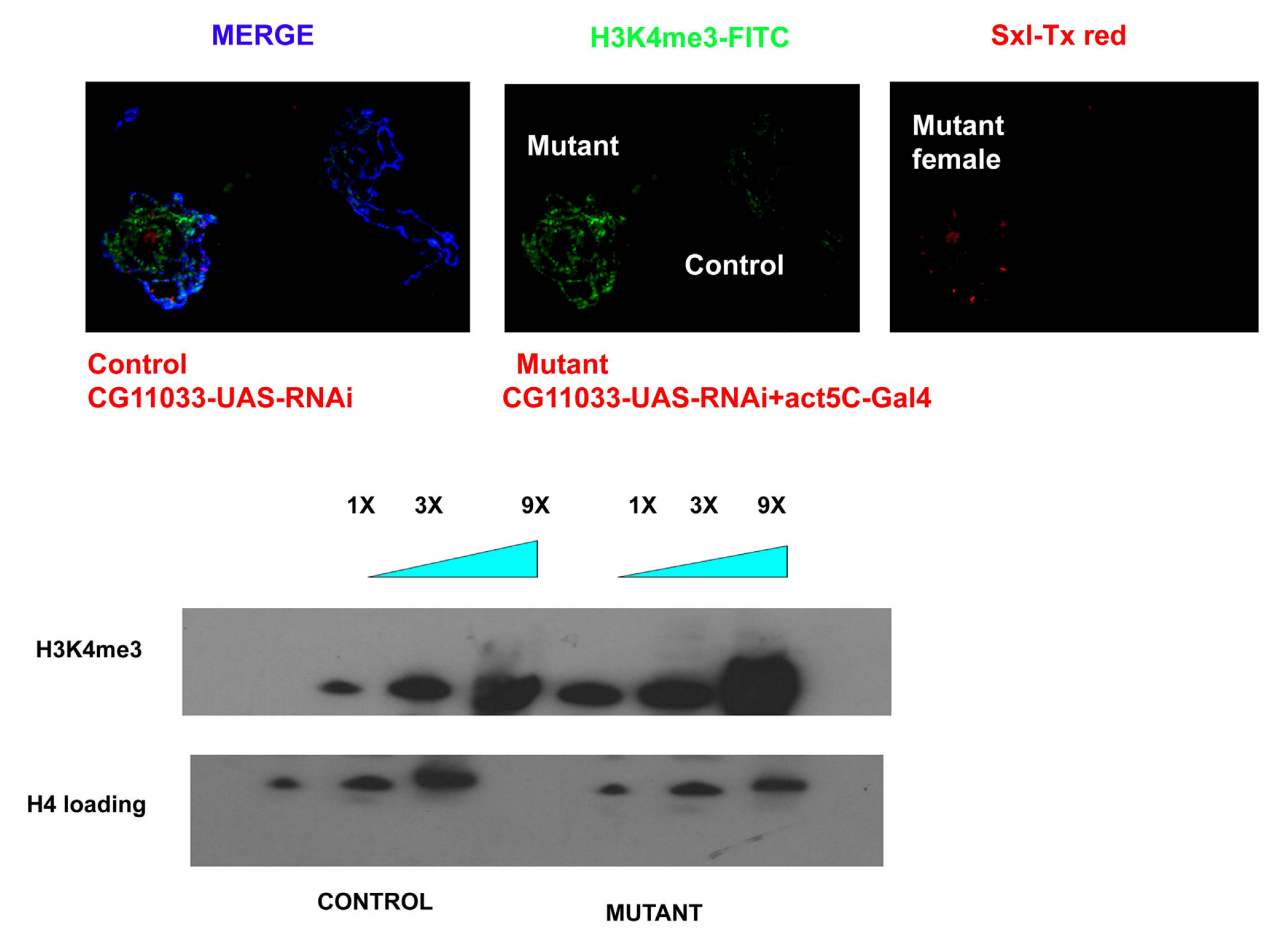
**Immunofluoresence analysis of larvae**. Mixtures of mutant and control larvae of different sexes were used. Sex lethal (Tx red) is used to distinguish male and female nuclei. H3K4me3 is stained by FITC. About 50 such pairs of nuclei were observed and a representative image is shown. Acid extracted histones from larvae were used for western blotting. CG11033-UAS-RNAi larvae (no Gal4) were used as controls while larvae expressing act5c-Gal4 were used as knockdown mutants.

**Figure 3:**
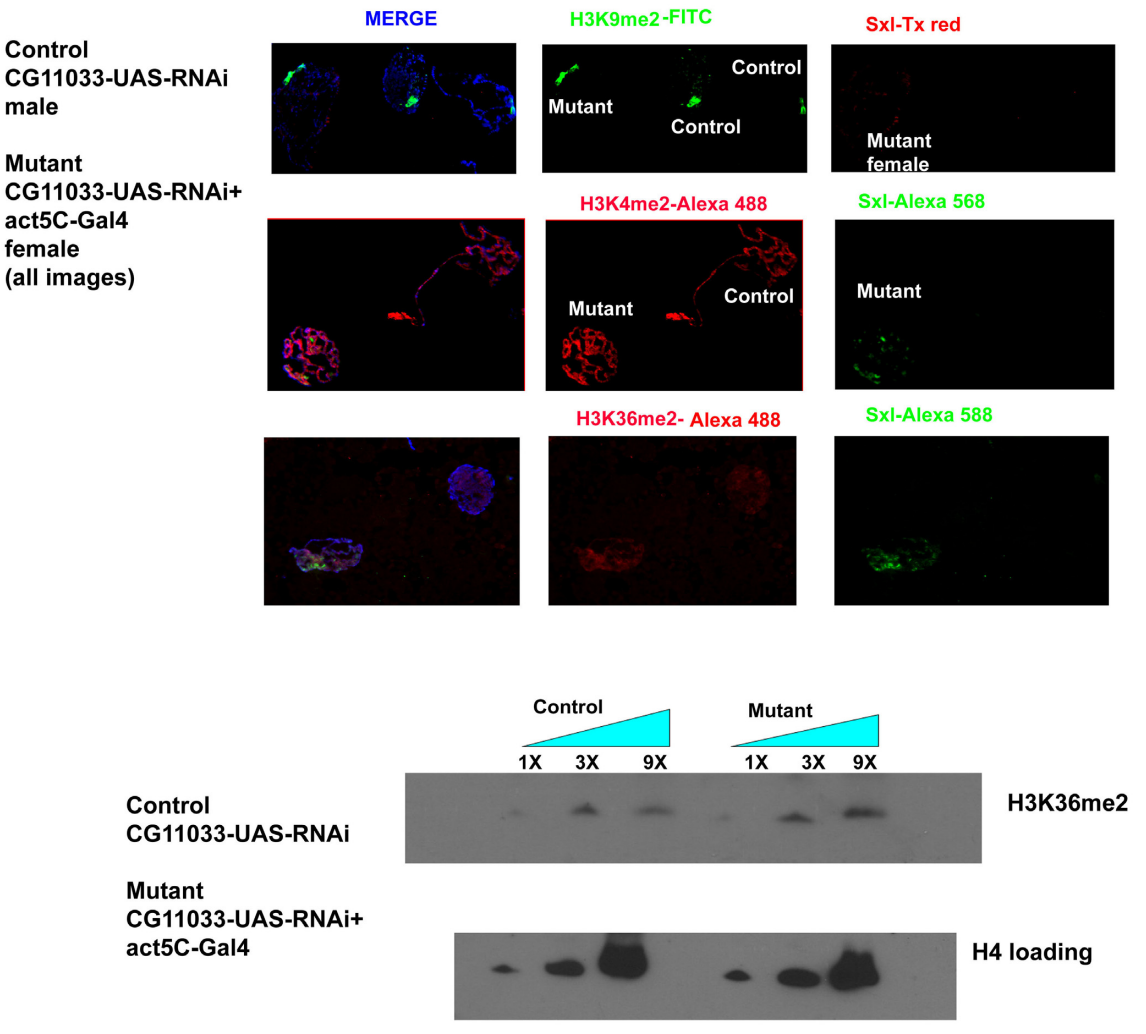
**Analysis of histone modifications in the dKDM2 RNAi knockdown mutants**. The RNAi knockdown dKDM2b mutants does not show any change in H3K36me2 as shown by Western blot as well as by immunostaining of polytene chromosomes. Similarly, no changes were detected in H3K4me2 and H3K9me2 modifications.

We then proceeded to determine if CG11033 knockdown affects histone modifications in vivo. The analysis of polytene chromosomes and western blot analysis of acid extracted histones from 3^rd ^instar larvae showed an increase in H3K4me3 modification in mutants compared to the control flies (Figure 2 and Additional file 5). For the immunofluoresence analysis we used a mixture of mutant and control nuclei in the same microscopic field. Antibodies against Sxl (Sex lethal), which is expressed only in the females, was used to distinguish between mutant and control nuclei. We could not detect any change in H3K36me2 levels by western blots or immunofluoresence analysis (Figure 3). Similarly no change was detected in the H3K4me2 pattern.

Because H3K4me3 modification is the site of active transcription, we examined whether a change in H3K4me3 status has any effect on H3K9me2 modification associated predominantly with silent heterochromatin. There was no observable change in H3K9me2 deposition at the chromocenter as visualized by the immunofluoresence staining of polytene chromosomes (Figure 3). Thus, the effect of CG11033 appears to be specific for regulating the H3K4me3 mark on the histones. The paradox wherein a genetically classified trithorax-like mutant, which removes H3K4me3 enhances PEV and decreases H3-acetyl marks, has been attributed to the titration of certain chromatin remodelers such as CHD1 [6]. In the usual situation, CHD1 binds to H3K4me3, but when H3K4me3 increases in *lid *mutants, CHD1 is titrated away from its usual genomic loci to other sites where it is usually not found, thus affecting transcriptional outcome. *Lid *has been shown to associate with RPD3 histone deacetylase and inhibit the biochemical activity of the later, thus, exerting a positive influence on transcription[7,8]. It will be interesting to explore whether dKDM2 is similarly associated with a histone deacetylase as part of a specialized protein complex. Thus, this study characterizes CG11033, which can be referred to as dKDM2 as a second H3K4me3 demethylase in addition to *lid*. Our study identified H3K4me3 as the histone methyl mark is strongly affected by this knockdown and not H3K36me2 as previously reported. In this context a recent study identified CG11033 (dKDM2) as a H3K36me2 specific demethylase in Drosophila cell lines [9]. However, dKDM2 can be affecting H3K4me3 under specific circumstances. In a recent study performed in mouse embryonic fibroblasts KDM2B has been shown to have demethylase activities towards both H3K4me3 and H3K36me2 [10]. The possibility that dKDM2 (like LSD1) could be interacting with other proteins in a context dependent manner to regulate H3K4me3 would be interesting to explore. Also unlike Lagarou et al. [9], our studies were performed in adult flies and not cell lines which could possibly explain different specificities of the enzyme (as a part of specific multiprotein complexes) towards the histone substrates. The inability to observe any effect on H3K36me2 in our experiments could also be attributed to incomplete knockdown of dKDM2/CG11033. In agreement with the mammalian studies, CG11033 (dKDM2) affects nucleolar organization and H3K4me3 modification. The transcription of tandem ribosomal RNA repeats is a tightly coordinated set of events, which ensures there is a formation of a single nucleolus. Recent studies have implicated epigenetic events in regulating the transcriptional regulation of rDNA repeats. Mutations in the RNAi machinery and Su(var) 3-9 bring about reduction in H3K9me2 at rDNA repeats and results in multiple nucleoli[11]. Because H3K4me3 is an important epigenetic landmark for active transcription, failure to erase this mark can result in aberrant transcription of rDNA repeats and failure to form a single compact nucleolus.

## Conclusion

The present study identifies dKDM2 (CG11033) as a novel JmjC domain histone lysine demethylases with specificity for H3K4me3 modification in vivo. The enzyme plays an important role in nucleolar structure organization.

## Materials and methods

### Fly crosses

RNAi strains were ordered from the VDRC (Vienna Drosophila Research Center). CG11033-UAS-RNAi females were crossed to act5c-Gal4/Tubby, Tb males. Non-tubby flies were used as knockdowns expressing the RNAi construct.

### Immunofluoresence analysis of polytene chromosomes

Third instar larvae expressing both Gal4 and the RNAi construct (non-Tubby) were selected as mentioned above. Larvae containing only the hairpin construct were used as controls (No Gal4). Three to four pairs of salivary glands each from control and mutants were dissected in 0.7% NaCl. The glands were then fixed for about a minute in 3.7% formaldehyde in PBS (ice-cold). The glands were then kept in a solution of 45% acetic acid and 3.7% formaldehyde for about 2 min and then squashed. The slide was kept on dry-ice for 20 min and then washed twice in phosphate buffered saline (PBS) (pH 7.4 containing 137 mM NaCl, 2.7 mMKCl and 0.0086 M dibasic potassium phosphate, 0.0015 M monobasic potassium phosphate) for 10 min each and blocked for 30 min in a solution of PBS containing bovine serum albumin (BSA). The following antibodies were used at 1:100 dilutions:Sxl (Hybridoma bank, University of Iowa), H3K4me3 (Active Motif) and H3K36me2, H3K4me2 and H3K9me2 (Upstate). The primary antibodies were incubated overnight at 4°C. The following day slides were washed twice in PBS and blocked in PBS-BSA solution. The slides were then blocked with 5% goat serum for 30 min at 37°C. The secondary antibodies (1:100 goat anti rabbit-FITC or goat anti mouse-Alexa 588 and 1:200 goat anti-mouse Texas red or goat anti-rabbit Alexa 488) were then applied to the slide for about 1 hr at 37°C. The slides were washed twice in PBS and visualized under fluorescence microscopy after application of DAPI (4',6-diamidino-2-phenylindole). The images were adjusted using Photoshop CS3 version software. The individual images in red and green channels were obtained by adjusting signal intensities of the original RGB image using channels option in the Photoshop CS3 version software. The red channel images were obtained by decreasing the blue and green channel signal intensities to zero. Similar adjustment was applied to obtain images in the green channel.

### Nucleolus staining

The gently squashed polytene spreads were prepared in the same manner except that solution II (45% acetic acid and PBS) was omitted. The glands were squashed in 0.7% NaCl and the fixed in 3.7% formaldehyde for 20 min at 4°C. The fibrillarin antibody (Abcam) was used at 1: 50 dilution.

### Western Blot analysis

Third instar larvae (12-15) were were homogenized in HEPES buffer containing protease inhibitor cocktail (Pierce). The homogenate was then acidified with HCl to a final concentration of 0.2 N HCl and kept on ice for 1 hr. The homogenate was then centrifuged at 11,000 rcf for 15 min and the supernatant was then neutralized with NaOH. The histone enriched protein lysate was then boiled with Laemmli sodium dodecyl sulfate (SDS) sample buffer and loaded on to the gel. The western blot analysis was performed by standard method as described in AbcamInc.protocols. The antibodies used were rabbit polyclonal H3K4me3 (1:1000), H3K36me2 (1:1000) and H4 loading control (1:1000). Pierce super signal picochemiluminescent substrate kit was used to observe the bands and Image gauge software was used to measure the density of bands.

### RNA extraction, cDNA synthesis and Real Time PCR analysis

RNAi knockdown larvae were Act5C-Gal4 + UAS-CG11033 inverted repeat) and control larvae were UAS-CG11033 inverted repeat (No Gal4). RNA was extracted from 3^rd ^instar larvae using Trizol reagent (Invitrogen Inc.). One microgram of RNA from control and RNAi knockdown mutant each was digested with DNAse I (Invitrogen). cDNA synthesis was performed using oligo (dT) primers and Superscript III Reverse transcriptase system (Invitrogen Inc.). ABI 7300 (Applied Biosystems Inc.) real time PCR machine was used for quantification of mRNA using SYBR green mix dye (Applied Biosystems Inc.). Dissociation curve was performed to establish the specificity of primers used in the experiment. Tubulin was used as an internal control and the relative mRNA level was calculated using the difference in Ct values between control and RNAi knockdown flies.

## Competing interests

The authors declare that they have no competing interests.

## Authors' contributions

HHK performed the experiments; HHK and JB composed the paper. The authors have read and approved the final manuscript.

## Supplementary Material

Additional file 1 **Amino acid sequence of dKDM2**. Amino acid sequence of CG11033 (dKDM2). The consensus nucleolar sequence is highlighted in red. Click here for file

Additional file 2 **Analysis of nucleoli in wild type and RNAi-CG11033 (dKDM2)**. Analysis of polytene nuclei with multiple nucleolar spots between wild type and RNAi-dKDM2 (CG11033). RNAi knockdown mutants show a greater frequency of multiple nucleoli compared to wild type. Click here for file

Additional file 3 **Real time PCR data analysis of CG11033 (dKDM2) mRNA**. Real time PCR data showing reduction in relative mRNA of dKDM2 (CG11033) in RNAi knockdown vs control (no RNAi) 3^rd ^instar larvae. Click here for file

Additional file 4 **Real time PCR data analysis of *lid *mRNA**. Real time PCR data showing no significant change in relative mRNA of *lid *in RNAi knockdown vs control (no RNAi) 3^rd ^instar larvae. Click here for file

Additional file 5 **Western blot analysis of H3K4me3 levels**. Levels of H3K4me3 between RNAi knockdown mutants of CG11033 (dKDM2) and control from four different experiments. The RNAi knockdown mutants show significantly higher level of H3K4me3.Asterisk indicates p value < 0.05. Click here for file

## References

[B1] Tsukada Y, Fang J, Erdjument-Bromage H, Warren ME, Borchers CH, Tempst P, Zhang Y (2006). Histone demethylation by a family of JmjC domain-containing proteins. Nature.

[B2] Koyama-Nasu R, David G, Tanese N (2007). The F-box protein Fbl10 is a novel transcriptional repressor of c-Jun. Nat Cell Biol.

[B3] Sánchez C, Sánchez I, Demmers JA, Rodriguez P, Strouboulis J, Vidal M (2007). Proteomics analysis of Ring1B/Rnf2 interactors identifies a novel complex with the Fbxl10/Jhdm1B histonedemethylase and the Bcl6 interacting corepressor. Mol Cell Proteomics.

[B4] He J, Kallin EM, Tsukada YI, Zhang Y (2008). The H3K36 demethylase Jhdm1b/Kdm2b regulates cell proliferation and senescence through p15(Ink4b). Nat Struct Mol Biol.

[B5] Frescas D, Guardavaccaro D, Bassermann F, Koyama-Nasu R, Pagano M (2007). JHDM1B/FBXL10 is a nucleolar protein that represses transcription of ribosomal RNA genes. Nature.

[B6] Eissenberg JC, Lee MG, Schneider J, Ilvarsonn A, Shiekhattar R, Shilatifard A (2007). The trithorax-group gene in Drosophila *little imaginal discs *encodes a trimethylated histone H3Lys4 demethylase. Nat Struct Mol Biol.

[B7] Lloret-Llinares M, Carré C, Vaquero A, de Olano N, Azorín F (2008). Characterization of *Drosophila melanogaster *JmjC+N histone demethylases. Nucleic Acids Research.

[B8] Lee N, Erdjument-Bromage H, Tempst P, Jones RS, Zhang Y (2009). The H3K4 demethylase Lid associates with and inhibits the Histone Deacetylase Rpd3. Molecular and Cellular Biology.

[B9] Lagarou A, Mohd-Sarip A, Moshkin YM, Chalkley GE, Bezstarosti K, Demmers JA, Verrijzer CP (2008). dKDM2 couples histone H2A ubiquitylation to histone H3 demethylation during Polycomb group silencing. Genes and Development.

[B10] Tzatsos A, Pfau R, Kampranis SC, Tsichlis PN (2009). Ndy1/KDM2B immortalizes mouse embryonic fibroblasts by repressing the Ink4a/Arf locus. Proc Natl Acad Sci.

[B11] Peng JC, Karpen GH (2007). H3K9methylation and RNA interference regulate nucleolar organization and repeated DNA stability. Nat Cell Biol.

