# Supplementary material for: Drosophila KDM2 is a H3K4me3 demethylase regulating nucleolar organization
Source: BMC Res Notes. 2009 Oct 23;2:217. doi: 10.1186/1756-0500-2-217 (PMC2771041; doi:10.1186/1756-0500-2-217)
Supplement: Additional file 1 — Amino acid sequence of dKDM2. Amino acid sequence of CG11033 (dKDM2). The consensus nucleolar sequence is highlighted in red. [file 1756-0500-2-217-S1.DOC]

GSVIGMGAGV EYSNGVM KKEQLENGSGVTV GGHGSQPEAT FALPTDTLKY RPPKKMHLATALVAAAASSS SGGGGPVAGV GGSAVVGSSH SPTGGGVGPV TGAGGAISVI ATSSSYIEGGCHSPEGGGAKLSPNLTGTGQ PRRRRTRCKN CAA

CQRSDCG TCPFCMDMVKFGGPGRAKQTCMMRQCLSPMLPVTAQCVYHLD

GWRQTPVSPQTKQLASA DGPSALMECSVCYEIAHPDC ALSQLDGTED AADA

KGIVNEDLPNSWECPS CCRSGKNYDY KVRLPSTHVSLVLVQLTNPT FIVQPRH

FRARQKSSEVRRVSVSHGQGGAEGHADGNTLLPPPVGQYNDFVFTSESEME

SGTVSGHMTHWKHGMKRHHQLEVKTERNNSCDTPSPGISPNA IGGDSKVG

**KRRK**SDDGTSVS SSMHESNDAPCGSSAEGAGGAGNANVSTNQWSGSGGG

GGSRKKNSIRSQL AQQMLNSSTPVLKKPQYVVRPASGTGSSSS SGNGGSASAT NGISNGSNQSGANSCGAGNG ERGTNNGGLS GSNGLGNQHY SSSQNLALDPTV

LKIIFRYLPQDTLVTCCSVCKVWSNAAVDPDLWKKMNC SEHKMSASLL TAIV

RRQPEHLILDWTQIAKRQLAWLVARLPALKNLSLQNCPIQAVLALH TCLCPPLQTL DLSFVRGLND AAIRDILSPP KDSRPGLSDSKTRLRDLKVM KLAGTDISDVAVRYITQS

LPYLRHLDLSSC QRITDAGVAQ IGTSTTATARLTELNLSACRLVSENALEHL AKCEGLI

WLDLRHVPQVSTQSVIRFASNSKHDLCVRDIKLVE**RRRR**NSTTANRSWHHD

**CONSENSUS NoLS- R/K-R/K-X-R/K highlighted in red**

Additional file 1. Amino acid sequence of CG11033 (dKDM2). The consensus nucleolar sequence is highlighted in red.
