# Supplementary material for: Drosophila KDM2 is a H3K4me3 demethylase regulating nucleolar organization
Source: BMC Res Notes. 2009 Oct 23;2:217. doi: 10.1186/1756-0500-2-217 (PMC2771041; doi:10.1186/1756-0500-2-217)
Supplement: Additional file 2 — Analysis of nucleoli in wild type and RNAi-CG11033 (dKDM2). Analysis of polytene nuclei with multiple nucleolar spots between wild type and RNAi-dKDM2 (CG11033). RNAi knockdown mutants show a greater frequency of multiple nucleoli compared to wild type. [file 1756-0500-2-217-S2.DOC]

Additional file 2. Analysis of polytene nuclei with multiple nucleolar spots between wild type and RNAi-dKDM2(CG11033).
