# Supplementary material for: Drosophila KDM2 is a H3K4me3 demethylase regulating nucleolar organization
Source: BMC Res Notes. 2009 Oct 23;2:217. doi: 10.1186/1756-0500-2-217 (PMC2771041; doi:10.1186/1756-0500-2-217)
Supplement: Additional file 4 — Real time PCR data analysis of lid mRNA. Real time PCR data showing no significant change in relative mRNA of lid in RNAi knockdown vs control (no RNAi) 3rd instar larvae. [file 1756-0500-2-217-S4.DOC]

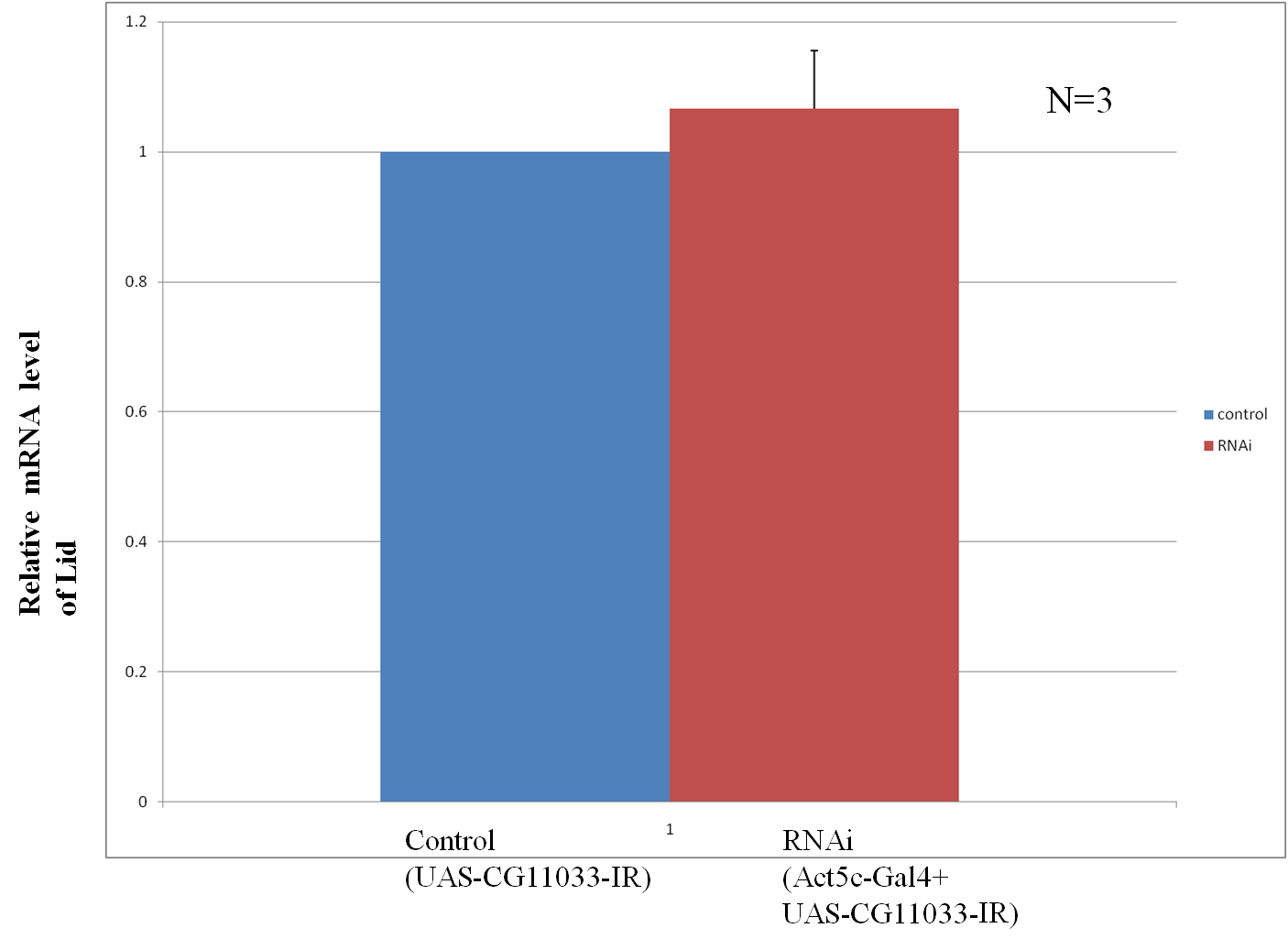


Additional file 4

Real time PCR data showing no significant change in relative mRNA of *lid* in RNAi knockdown vs control (no RNAi) 3^rd^ instar larvae.
