# Supplementary figures and images for: Drosophila KDM2 is a H3K4me3 demethylase regulating nucleolar organization
Source: BMC Res Notes. 2009 Oct 23;2:217. doi: 10.1186/1756-0500-2-217 (PMC2771041; doi:10.1186/1756-0500-2-217)

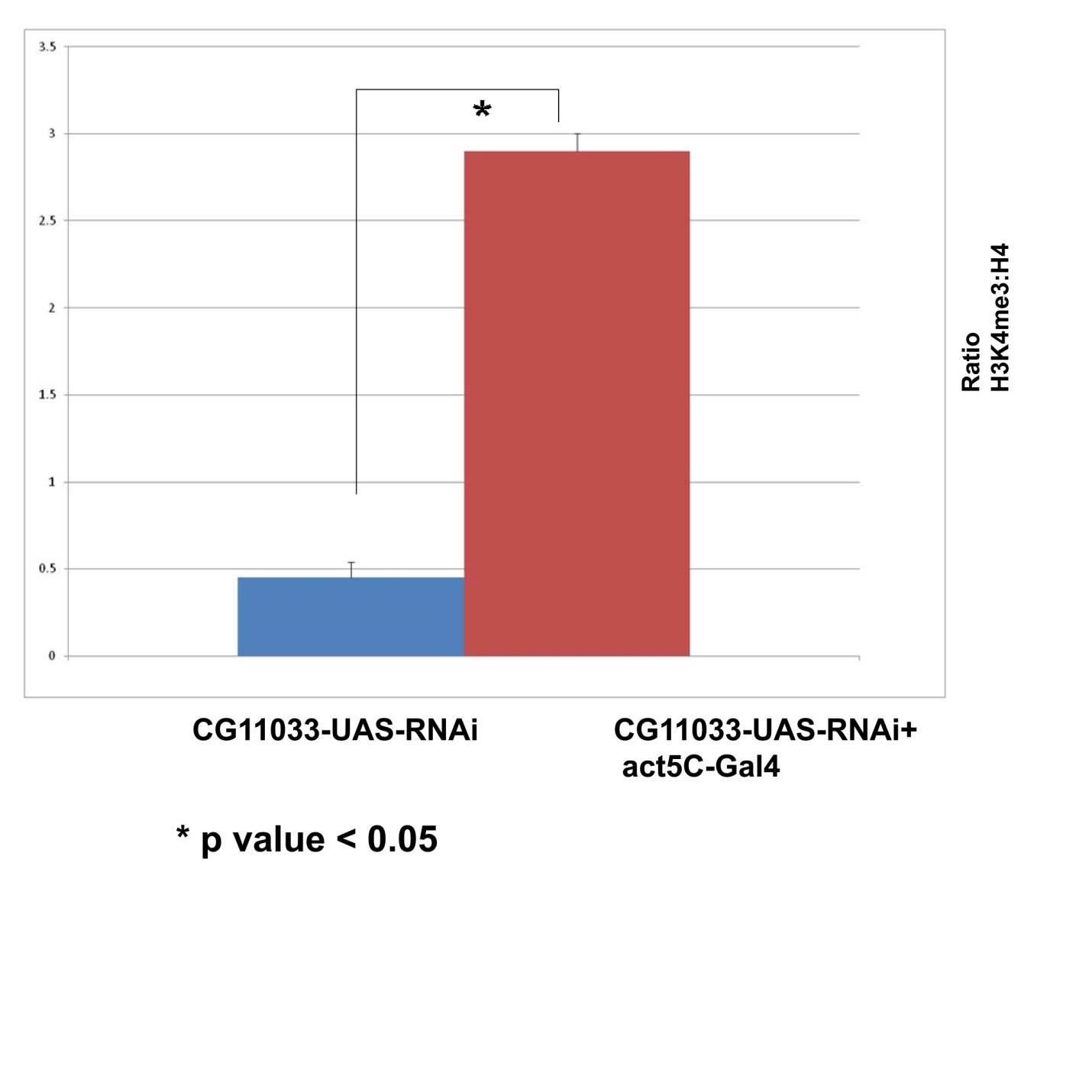


Additional file 5

Western blot analysis of H3K4me3 levels between wild type and RNAi-dKDM2 (CG11033).

Supplement: Additional file 5 — Western blot analysis of H3K4me3 levels. Levels of H3K4me3 between RNAi knockdown mutants of CG11033 (dKDM2) and control from four different experiments. The RNAi knockdown mutants show significantly higher level of H3K4me3.Asterisk indicates p value < 0.05. [file 1756-0500-2-217-S5.DOC]
